# Extract from the Minutes of the Georgia Medical Association

**Published:** 1872-03

**Authors:** 


Extract from the minutes of the Georgia Medical Association, at its meeting in Americus, April, 1871:

"Whereas, The controversies between the Faculty of the Atlanta Medical College, the Atlanta Academy of Medicine, the Fulton County Medical Society, or members of the same, who are members of this body, or between said bodies or individuals, and other members of this Association, are of a personal character, and ought never to have been introduced into the Association; therefore,

"*Resolved,* That all action of the Association upon these controversies be rescinded, and be no longer regarded as a part of the archives of this body."

This disposition of the difficulties which had been introduced into the Association was made by an overwhelming majority (more than two-thirds of the body), and should afford a guaranty to all, who desire to attend the meeting in Columbus, that there is no risk of being entangled in further troubles.

